# Effect of the SAFE-CARE nursing bundle on artificial airway-related complications in adult ICU patients: a before-and-after interrupted time-series study

**DOI:** 10.3389/fmed.2026.1802134

**Published:** 2026-03-25

**Authors:** Yayun Qin, Li Gao, Yuhe Jin, Xinxin Li, Juan Wang

**Affiliations:** Department of Intensive Care Unit, The Fifth Affiliated Hospital of Anhui Medical University/The Fuyang Affiliated Hospital of Anhui Medical University, Fuyang, Anhui, China

**Keywords:** ICU - intensive care unit, interrupted time series (ITS), pericuff microaspiration, SAFE-CARE, VAP (ventilator-associated pneumonia)

## Abstract

**Objective:**

To evaluate the effectiveness and sustainability of the SAFE-CARE nursing bundle in reducing artificial airway related complications in adult ICU patients, and to assess whether it provides a practical model for low and middle income countries and under resourced ICUs.

**Methods:**

This single center retrospective before and after interrupted time series study included adult patients who received invasive mechanical ventilation with an artificial airway retained for at least 24 h. The control group was February to December 2023 and the intervention group was January to December 2024. Primary outcomes included pericuff microaspiration (PM), ventilator associated pneumonia (VAP), medical device related pressure injury (MDR-PI), and unplanned extubation (UE). Secondary outcomes included duration of mechanical ventilation, ICU length of stay, and nursing checklist compliance.

**Results:**

A total of 122 patients were included, with 61 patients in each group. PM decreased from 29.5 to 6.6%, VAP decreased from 26.2 to 4.9%, MDR-PI decreased from 19.7 to 4.9%, and UE decreased from 31.1 to 3.3%. Mechanical ventilation duration and ICU length of stay showed no significant differences between groups. Checklist compliance improved from 67.9 to 90.80%. Interrupted time series analysis showed an immediate reduction after implementation, with sustained post implementation declines for PM and VAP.

**Conclusion:**

SAFE-CARE was associated with rapid and clinically meaningful reductions in multiple airway related complications and demonstrated sustained improvement for key infection related outcomes, suggesting a scalable low cost strategy for general ICUs.

## 1 Introduction

Ensuring a clear airway using endotracheal intubation or tracheostomy is a key procedure to support breathing in critically ill patients. However, having an artificial airway can reduce the effectiveness of coughing, weaken mucociliary clearance, and numb the throat area. These effects increase the risk of serious complications.

A meta-analysis showed that 6.7% of adult intensive care unit (ICU) patients with intubation experienced unplanned extubation (UE), most of which were self-initiated (1). UE is not harmless—it has been linked to higher rates of ventilator associated pneumonia (VAP), longer ICU and hospital stays, and increased death rates (2). Meanwhile both lab and clinical studies confirm that microaspiration occurs in up to 88% of intubated ICU patients (3). The leakage of bacteria-laden fluids from the mouth and stomach into the lungs is the main pathway for infection (4). Since microaspiration usually has no visible signs, it has been called the “silent enemy” of mechanical ventilation. Medical device related pressure injuries (MDR-PIs) are also common. Airway devices are the main cause: one review found they are a frequent source of such injuries in ICU patients (5). A large European study reported a 36.8% rate of MDR-PIs during the first 8–11 days of ICU stay (6). Another study estimated that up to 68% of these injuries are related to respiratory devices (7).

Although guidelines recommend prevention bundles targeting modifiable risks, there is no single best practice (8). To reduce these complications, many researchers support multi-part care bundles. A systematic review of 38 studies reported that 22 studies achieved >36% VAP reduction, and 10 studies achieved >65%, with bundles commonly including head-of-bed elevation and cuff pressure control (9). However, two main problems remain. First most interventions focus on just one issue, not the combined risks of UE, microaspiration, VAP, and MDR-PI. Second many bundles rely on costly devices like subglottic tubes, automated cuff pumps, or electronic monitoring systems, which are not available in low-resource hospitals.

To address these gaps, we created SAFE-CARE—a low-cost, nurse-led care bundle. It includes secure tube fixation, regular manual cuff pressure checks, oral and subglottic cleaning, head-up positioning, two-hourly patient repositioning with foam padding, daily sedation breaks, and a checklist with feedback. In this single-center, retrospective interrupted time-series study, we compare the rates of pericuff microaspiration (PM), VAP, MDR-PI, and UE before and after SAFE-CARE was put into practice. We hypothesize that this manual, evidence-based approach can significantly lower all four complications and offer a practical model for general ICUs without advanced equipment.

## 2 Materials and methods

### 2.1 Study design

This was a single-center, retrospective before after study using an interrupted time-series design. The study was conducted in the general ICU of The Fifth Affiliated Hospital of Anhui Medical University. Patients admitted between February, 2023 and December, 2024, were evaluated. A structured care bundle, SAFE-CARE, was formally implemented on January 1, 2024. Accordingly, the period from February to December, 2023 was defined as the control phase, and January, 2024 to December, 2024 was defined as the intervention phase. The study aimed to assess the effect of SAFE-CARE on airway-related complications.

The intervention evaluated in this study was the formal ICU-wide implementation of the SAFE-CARE bundle on January 1, 2024. This date corresponded to the start of standardized bedside checklist use, structured staff training, routine fidelity auditing, and monthly audit-feedback reporting. Although some individual airway-care practices may have existed in routine care before this date, they had not previously been implemented as a unified bundle with predefined frequencies, standardized documentation, and compliance monitoring. Therefore, January 1, 2024 was treated as the intervention point in the interrupted time-series analysis.

### 2.2 Study population

Eligible patients were those who received invasive mechanical ventilation and retained an artificial airway for ≥24 h during the specified study period. Patient information was extracted from the hospital’s electronic medical record system. Nursing records were obtained through the hospital’s integrated nursing information system to ensure data completeness and traceability.

### 2.3 Inclusion criteria

Patients were included if they met all of the following criteria: (1) age ≥18 years; (2) received oral, nasal, or tracheostomy airway intubation, with airway retention >24 h; (3) intact oral, nasal, and cervicofacial skin at ICU admission; (4) Braden score ≤12 on ICU admission; and (5) complete clinical and nursing records, with no missing key variables.

### 2.4 Exclusion criteria

Patients were excluded if they met any of the following conditions: (1) pre-existing MDR-PI at ICU admission; (2) Richmond Agitation–Sedation Scale (RASS) score ≤ − 4 or ≥ + 3, indicating severely abnormal consciousness; (3) expected airway removal or ICU discharge within 24 h; or (4) incomplete medical data or outcome variables preventing full evaluation.

### 2.5 Grouping method

This study employed a natural grouping approach. Patients admitted before January, 2024—prior to the implementation of the SAFE-CARE care bundle—were assigned to the control group. Those admitted on or after this date were assigned to the intervention group. For patients whose hospitalization spanned the implementation date, group assignment was based on the first 48 h of ICU admission, in order to minimize bias and avoid contamination from mixed care practices (Table 1).

**Table 1:** Key components of the SAFE-CARE bundle (requires only routine consumables).

Module	Detailed procedures
S—Secure fixation “Safe tube stabilization”	1. For endotracheal tubes: use cross and spiral taping, cover with 3 M transparent film twice; verify tube depth and bilateral breath sounds every shift.
	2. For tracheostomy: change dedicated tie daily; tension should allow insertion of 1–2 fingers; after 24 h, apply a thin transparent dressing between tie and neck skin.
	3. Assess tube position and skin integrity each shift; adjust angle or fixation side to avoid shear/friction.
	4. Apply foam/silicone pads to high-risk areas; use lip or nasal cushions when needed.
A—Airway cuff manual check “Cuff pressure monitoring”	1. Maintain cuff pressure at 25–30 cmH₂O; check every 6–8 h manually.
	2. Recheck immediately after repositioning, suctioning, or positive end-expiratory pressure (PEEP) adjustment.
	3. If pressure is above target, deflate slowly from 2 cmH₂O above setpoint to prevent rebound.
	4. Drain condensate regularly from the pressure line.
	5. For alert invasive patients, consider deflation or use of cuffless tubes.
F—Frequent oral & subglottic care “High-frequency oral/supraglottic care”	1. Oral care every 6–8 h: brush and rinse; avoid chlorhexidine, use saline or non-antiseptic mouthwash.

	2. Intermittent subglottic suction every 1–2 h; if no port, use two-nurse “PEEP-flush” technique to clear secretions above the cuff.
	3. Suction oral and supraglottic secretions before repositioning.
	4. Suction oral/pharyngeal secretions at least every 4 h.
E—Elevated position “Head-up + reflux prevention”	1. Elevate head of bed to 30°–45° if clinically feasible.
	2. Use nasogastric tube feeding with controlled drip rate; check gastric residual every 4 h; pause if >200 mL.
	3. Add prokinetics and monitor intra-abdominal pressure if needed.
	4. Maintain PEEP at 5 cmH₂O to prevent secretion backflow.
C—Checklist audit “Audit-feedback mechanism”	1. Use bedside checklist of 16 evidence-based items (e.g., risk assessment, cuff management, skin fixation, oral care).
	2. Quality control nurses audit ≥20% of records weekly; initiate PDSA if compliance <80%.
	3. Publish audit results monthly and incorporate into staff education.
A—Awaken daily “Daily sedation interruption”	1. Target sedation to RASS −1 to 0.
	2. Perform daily sedation hold when not contraindicated to assess spontaneous breathing and extubation readiness.
	3. For patients with extubation attempts, apply light restraints and provide verbal reassurance.
R—Rotate & relieve pressure “Turning and pressure offloading”	1. Reposition every 2 h + perform chest physiotherapy.
	2. Assess skin at oral/nasal/neck/stoma sites each shift; change fixation point or side if needed.
	3. Maintain tracheostomy tube in neutral position; use ventilator arm to reduce traction.
	4. Use foam dressings for high-risk skin; switch to smaller tubes or softer cuffs if needed.
E—Education “Continuous staff and patient education”	1. Provide 3-tier training: onboarding → quarterly evaluation → case review.
	2. Post SAFE-CARE flowchart at bedside; prepare 1-min QR-coded microlearning videos.
	3. Educate patients/families on airway importance, nonverbal communication, and anxiety/extubation risk reduction.

### 2.6 Outcome measures and definitions

Pericuff microaspiration surveillance was performed according to the ICU monitoring protocol and was defined as *α*-amylase >2000 U/L in tracheal aspirates or a positive modified Evans blue dye test. Measurements were recorded twice daily while the artificial airway remained in place, and outcome data were extracted from contemporaneously documented clinical records (Table 2).

**Table 2:** Outcome measures and definitions.

Category	Indicator	Definition criteria
Primary outcomes	1. PM rate	Defined as α-amylase >2000 U/L or a positive modified Evans blue dye test; measured twice daily.
	2. VAP	Diagnosed based on the “Diagnostic Criteria for Nosocomial Infections” (10).
	3. MDR-PI	Graded using the EPUAP classification system; Stage I and above were recorded.
	4. UE	Included any unintended tube dislodgement or patient self-extubation.
Secondary outcomes	1. Duration of mechanical ventilation	Automatically extracted from the hospital’s electronic medical record system.
	2. ICU length of stay	
	3. Nursing checklist compliance	

VAP events were identified from the hospital infection surveillance platform and cross-checked against medical records, microbiological findings, and radiographic documentation. Case ascertainment was based on the institutional nosocomial infection diagnostic criteria, consistent with guideline-based VAP definitions (10), namely pneumonia occurring >48 h after endotracheal intubation or tracheotomy for mechanical ventilation, or within 48 h after ventilator withdrawal and extubation, together with a new or progressive infiltrate, consolidation, or ground-glass opacity on chest imaging plus at least two of the following: fever >38 °C, purulent airway secretions, or a peripheral white blood cell count >10 × 10^9^/L or <4 × 10^9^/L.

MDR-PI was classified according to the EPUAP staging system based on routine nursing skin assessments and wound-related documentation. Because of the retrospective design, assessors were not blinded to study period; however, outcome abstraction was independently performed by two trained research nurses, with adjudication by a senior ICU nurse when discrepancies occurred.

UE included both patient self-extubation and accidental tube dislodgement. Events were identified from nursing adverse-event reports and time-stamped bedside records, and categorization was based on the contemporaneous event description in the original chart.

### 2.7 Data collection and quality control

Clinical and nursing data were retrieved from multiple platforms, including the ICU electronic medical record system, the nursing documentation system, the hospital infection surveillance platform, and internal quality control records. This multi-source integration enabled cross-validation and improved data fidelity. Two designated research nurses, trained under a unified protocol, independently collected and verified all primary data. In cases of inconsistency, a senior ICU nurse expert served as an adjudicator to ensure data integrity and consistency. When discrepancies occurred, the time-stamped primary source prevailed, and any remaining unresolved cases were adjudicated by a senior ICU nurse using original source documents, with all adjudication decisions documented. These procedures were implemented to minimize selection bias and enhance the robustness and generalizability of the final analysis. Implementation fidelity was monitored using a 16-item bedside checklist. Quality-control nurses audited at least 20% of records weekly, and monthly compliance summaries were fed back to frontline staff. When compliance fell below the predefined threshold of 80%, corrective actions were initiated using a Plan-Do-Study-Act approach. These measures were intended to reduce fidelity drift and support stable bundle implementation over time.

### 2.8 Statistical analysis

Statistical analyses were performed using SPSS version 27.0 and R version 4.3.2. Continuous variables were expressed as mean ± standard deviation (SD). Categorical variables were reported as absolute frequency (*n*) and percentage (%). *T*-tests and chi-square or Fisher’s exact tests were used for patient-level comparisons between the pre-intervention and post-intervention groups as two independent samples. These analyses were descriptive and did not account for time. To evaluate longitudinal trends and intervention effects, interrupted time-series (ITS) analysis with segmented regression was employed. Autocorrelation was assessed using the Durbin–Watson statistic, residual autocorrelation function (ACF), and Ljung–Box test. If autocorrelation was detected, ordinary least squares (OLS) regression with heteroscedasticity and autocorrelation consistent (HAC) standard errors was retained as the primary ITS model. In addition, a generalized linear model (GLM)-based segmented regression was performed as a sensitivity analysis to verify the robustness of the ITS findings. All statistical tests were two-sided, with a significance level set at *p* < 0.05.

## 3 Results

### 3.1 Primary outcomes

A total of 122 patients were included, with 61 patients in each group. The incidence of PM significantly decreased from 29.5% in the control group to 6.6% in the intervention group (χ^2^ = 10.87, *p* < 0.01), suggesting that the SAFE-CARE bundle may effectively reduce aspiration-related risks. The incidence of VAP also declined markedly from 26.2 to 4.9% (χ^2^ = 10.54, *p* < 0.01), indicating a strong preventive effect of the intervention on VAP.

The occurrence of MDR-PI dropped from 19.7 to 4.9% (χ^2^ = 6.16, *p* < 0.05), highlighting the bundle’s protective benefit on skin and mucosal integrity. Similarly, the rate of UE was reduced from 31.1 to 3.3% (χ^2^ = 16.62, *p* < 0.01), reflecting improved tube fixation and airway management following implementation of SAFE-CARE.

### 3.2 Secondary outcomes

The duration of mechanical ventilation was not significantly different between groups, 72.33 ± 47.79 h in the control group and 80.67 ± 72.20 h in the intervention group (*t* = 0.753, *p* = 0.453), suggesting no significant effect of the intervention on ventilation duration. Similarly, the ICU length of stay was not significantly different, 19.84 ± 16.65 days in the control group and 17.89 ± 12.58 days in the intervention group (*t* = 0.730, *p* = 0.467), indicating no significant difference in ICU hospitalization time between groups.

Lastly, the compliance rate with the SAFE-CARE checklist improved significantly, rising from (67.9 ± 11.0)% in the control group to (90.80 ± 6.69)% in the intervention group (*t* = 13.842, *p* < 0.01), reflecting enhanced adherence to nursing protocols and improved quality control performance (Table 3).

**Table 3:** Comparison of primary and secondary outcomes before and after SAFE-CARE implementation.

Outcome measure	Control group (*n* = 61)	Intervention group (*n* = 61)	Test statistic	*p*-value
PM, *n* (%)	18 (29.5%)	4 (6.6%)	10.869	0.001
VAP, *n* (%)	16 (26.2%)	3 (4.9%)	10.536	0.001
MDR-PI, *n* (%)	12 (19.7%)	3 (4.9%)	6.157	0.013
UE, *n* (%)	19 (31.1%)	2 (3.3%)	16.623	0.000
Duration of mechanical ventilation (hours)	72.33 ± 47.79	80.67 ± 72.20	0.753	0.453
ICU length of stay (days)	19.84 ± 16.65	17.89 ± 12.58	0.730	0.467
Nursing checklist compliance (%)^a^	67.90 ± 11.05	90.80 ± 6.69	13.842	0.000

a Compliance is calculated as the average daily completion rate per patient, analyzed using independent sample *t*-test.

Detailed effect size estimates for the cross-sectional before–after comparisons, including group-specific 95% confidence intervals, absolute risk differences, relative risks, odds ratios, and mean differences, are provided in Supplementary Table S1.

### 3.3 Interrupted time-series analysis

To further control for potential confounding factors such as long-term trends variation, an ITS analysis using segmented regression was conducted. The analysis included monthly incidence rates (per 100 intubation-days) of major airway-related complications over an 23-month period from February 2023 to December 2024.

The ITS model was specified as follows:


$$\begin{array}{l} Y_{t}=\beta_{0}+\beta_{1}\cdot {\text{TIME}}_{t}+\beta_{2}\cdot {\text{INTERVENTION}}_{t}\\ +\beta_{3}\cdot \text{TIME}\_\mathrm{POS}T_{t}+\varepsilon_{t} \end{array}$$

Where:

TIME_t_ represents the chronological month number starting from February 2023; INTERVENTION_t_ is a binary indicator (0 before January 2024, 1 from January 2024 onward); TIME_POSTt counts the months since intervention (0 before January 2024, increasing monthly afterward); ε_t_ is the error term (Table 4).

**Table 4:** Segmented regression effects of SAFE-CARE on primary outcomes per 100 intubation days.

Outcome	*β*₀ baseline level (95% CI)	*β*₁ baseline slope (95% CI)	*β*₂ immediate level change (95% CI)	*β*₃ post-intervention slope change (95% CI)	Adjusted *R*^2^
PM	6.98 (4.50 to 9.45)	+0.33 (−0.03 to +0.70)	−8.31 (−11.50 to −5.11)*	−0.55 (−1.04 to −0.07)*	0.84
VAP	6.18 (4.82 to 7.54)	+0.30 (+0.02 to +0.58)	−7.40 (−9.91 to −4.89)*	−0.53 (−0.84 to −0.23)*	0.84
MDR-PI	5.48 (3.06 to 7.90)	+0.04 (−0.32 to +0.39)	−3.76 (−6.88 to −0.63)*	−0.27 (−0.74 to +0.21)	0.68
UE	8.48 (6.14 to 10.82)	+0.17 (−0.17 to +0.52)	−8.86 (−11.88 to −5.84)*	−0.35 (−0.81 to +0.11)	0.88

* *p* < 0.05 for *β*₂ and *β*₃ (level and slope changes).

*β*₀ represents the estimated baseline level at month 0; *β*₁ is the monthly pre-intervention trend; *β*₂ is the immediate change at the point of intervention; *β*₃ is the change in slope post-intervention. For VAP, 95% CI and *p* values were calculated using Newey–West HAC standard errors with maxlag = 2 because residual autocorrelation was detected. For PM, MDR-PI, and UE, conventional OLS standard errors were used because residual autocorrelation diagnostics were not statistically significant.

For immediate effect (*β*₂), in the month of SAFE-CARE implementation, the incidence of PM dropped significantly by 8.31 cases per 100 intubation day (ID) (95% CI: −11.50 to −5.11), demonstrating a strong immediate effect. Similarly, VAP, MDR-PI, and UE rates declined immediately by 7.40, 3.76, and 8.86 cases per 100 ID, respectively. These reductions indicate that the intervention yielded rapid clinical benefits and that frontline implementation was effective in achieving early risk control.

For trend effect (*β*₃), Following the initial impact, PM and VAP showed statistically significant post-intervention slope changes, while MDR-PI and UE did not show statistically significant slope changes. Microaspiration incidence decreased steadily by −0.55 cases per 100 ID per month post-intervention. The monthly decline in VAP incidence was −0.53 cases per 100 ID, reflecting progressive improvement in nursing compliance and the sustained effects of training and audit-feedback mechanisms. For MDR-PI, the estimated monthly slope change was −0.27 cases per 100 ID, but it was not statistical significance differently, suggesting that sustained improvement may require additional measures or longer follow-up. Although a downward trend was also observed for UE, the slope did not reach statistical significance, suggesting that further improvement may depend on structural or system-level factors.

The models demonstrated good explanatory power, with adjusted *R*^2^ values ranging from 0.68 to 0.88. Residual autocorrelation was examined using Durbin Watson statistics in the OLS summaries, and Ljung Box tests were additionally applied for the outcomes fitted with ARIMA error models. These diagnostics provided no evidence of meaningful residual autocorrelation at the reported lags. Normality diagnostics were not fully consistent across models. Overall, the residual checks support the adequacy of the ITS specifications for inference. The segmented ITS plots and residual plots are shown in Supplementary Figures S1, S2. In addition, GLM-based segmented regression showed the same overall trend patterns, further supporting the robustness of the findings (Supplementary Table S2).

In summary, the SAFE-CARE low-cost care bundle demonstrated immediate reductions across all outcomes and sustained downward trends for PM and VAP. The results underscore the clinical value and sustainability of bundled interventions integrated with audit–feedback loops, particularly in complex care environments with limited resources.

## 4 Discussion

In this single-center retrospective interrupted time-series study, implementation of the SAFE-CARE bundle was associated with immediate reductions in PM, VAP, MDR-PI, and UE, with sustained post-intervention declines observed for PM and VAP. These findings suggest that a nurse-led bundle based on routine consumables, standardized bedside practice, and audit-feedback may improve several airway-related outcomes in a general ICU. However, the results should be interpreted as evidence of association rather than definitive proof of causality, because the study design remains vulnerable to unmeasured time-varying confounding. Mechanical ventilation duration and ICU length of stay were not significantly reduced, indicating that complication reduction did not necessarily translate into shorter resource use within the observed sample and follow-up period.

The VAP findings are clinically important and broadly consistent with the literature. Patients in low- and middle-income countries have been reported to face a substantially higher burden of VAP, and successful prevention programs in such settings depend on strategies that are affordable and feasible for frontline staff (11, 12). Against that background, the reduction in VAP observed after SAFE-CARE implementation is notable because the bundle did not rely on proprietary endotracheal tubes or automated cuff-pressure systems. This direction and magnitude of effect are also broadly coherent with prior bundle literature, especially reports emphasizing head-of-bed elevation, cuff-pressure control, and secretion management (9). Because VAP is associated with added expenditure, prolonged hospitalization, mortality, and antibiotic use (13–15), the observed reduction may also have practical implications for stewardship and resource use, although cost extrapolation across health systems should be interpreted cautiously. The improvement in checklist compliance from 67.9 to 90.8% may have contributed to these gains and is consistent with other studies indicating that audit-feedback and cyclical adaptation can improve bundle adherence and narrow the evidence-practice gap (16–18).

The reductions in UE and MDR-PI further strengthen the clinical relevance of the bundle. UE is associated with frequent re-intubation and subsequent respiratory harm (19), and the post-intervention UE rate of 3.3% compares favorably with both the pooled incidence reported by Li et al. and remained within the range reported across individual ICU cohorts (1, 20). The MDR-PI findings are also consistent with the other studies. Recent evidence indicates that device-related pressure injuries remain common in critically ill adults, while prevention knowledge and practice gaps still exist in bedside care (21, 22). In that context, the decline observed here is coherent with prior protocol-based data showing that structured nursing protocols including scheduled device repositioning and pressure off-loading measures can reduce airway device-related pressure injury (23). It is also plausible that fewer rescue procedures and fewer airway-related adverse events reduced additional device manipulation, although that pathway was not directly tested in the present study.

Several mechanisms may plausibly explain the observed pattern, but these should be regarded as hypothesis-generating rather than as effects directly demonstrated by the present data. The balance between adequate tracheal sealing and avoidance of cuff-related mucosal injury is central to cuff-pressure management (24), and cuff pressure can change after routine body repositioning, which supports repeated manual reassessment (25). Semi-recumbent positioning may reduce reflux and aspiration of contaminated secretions (26). PEEP support and daily awakening may also contribute to airway stability and preservation of protective reflexes (27, 28). Positioning-based interventions may also support physiological stability, as passive orthostatic positioning was safe and improved ventilatory mechanics in mechanically ventilated ICU patients (29). Because VAP is strongly linked to microaspiration of colonized secretions (30), frequent oral and supraglottic clearance offers a plausible explanation for the immediate reduction in PM and VAP, even though suction protocols alone do not eliminate aspiration risk (31). For MDR-PI, the observed reduction is biologically coherent with the established roles of sustained pressure, shear, and ischemia–reperfusion injury (32). Taken together, these considerations support the proposed “triangular stabilization” framework as a useful interpretive model linking mechanical sealing and drainage, physiological stability, and pressure redistribution. However, this framework should be presented as a hypothesis rather than as a mechanism established by the present study.

Several limitations should be considered. First, this was a retrospective single-center study, which limits external validity. Second, although interrupted time-series analysis is stronger than a simple before-after comparison, it remains vulnerable to time-varying confounding, and monthly severity indicators, sedation practice patterns, nurse-to-patient ratios, ventilator mode distributions, and concurrent infection-control or antibiotic-stewardship changes were not consistently available for adjusted modeling. Third, several outcomes were derived from routine clinical documentation, which introduces the possibility of outcome misclassification and observer bias, particularly because assessors were not blinded to study period. Fourth, only 23 monthly time points were available, limiting formal assessment of seasonality and more complex time-series structures. Fifth, no subgroup analyses were performed, and the inclusion criteria may have enriched the cohort for high-risk patients. Accordingly, the findings are best interpreted as supporting feasibility and clinical association rather than definitive effectiveness.

Despite these limitations, the study has practical implications. SAFE-CARE was implemented using routine consumables, manual monitoring, bedside checklists, and feedback cycles rather than expensive proprietary equipment. This implementation profile may be particularly relevant to general ICUs and under-resourced settings where advanced airway technologies are not readily available. Future work should test the bundle in multicenter prospective or hybrid effectiveness-implementation studies, incorporate fidelity tracking and severity adjustment, and evaluate cost-effectiveness more directly.

## 5 Conclusion

Based on 23 months of interrupted time-series data, SAFE-CARE was associated with immediate reductions in PM, VAP, MDR-PI, and UE, with sustained post-intervention declines observed for PM and VAP. Mechanical ventilation duration and ICU length of stay did not change significantly, whereas checklist compliance improved substantially after implementation.

These findings suggest that a nurse-led, low-cost airway care bundle built from routine consumables, standardized bedside procedures, and audit-feedback may offer a feasible strategy for reducing multiple airway-related complications in general ICUs, particularly in resource-constrained settings.

The proposed “triangular stabilization” framework may help interpret these findings, but it should be regarded as hypothesis-generating rather than as a mechanism proven by the present study. Future multicenter prospective studies with severity adjustment, fidelity measurement, and formal cost-effectiveness evaluation are needed.

## Data Availability

The original contributions presented in the study are included in the article/Supplementary material, further inquiries can be directed to the corresponding author.
