# Supplementary material for: Effect of the SAFE-CARE nursing bundle on artificial airway-related complications in adult ICU patients: a before-and-after interrupted time-series study
Source: Front Med (Lausanne). 2026 Mar 25;13:1802134. doi: 10.3389/fmed.2026.1802134 (PMC13057520; doi:10.3389/fmed.2026.1802134)
Supplement: Supplementary file 1 [file Table_1.DOCX]

**Table S1. Additional effect size estimates and 95% confidence intervals for before–after comparisons**

**Panel A. Dichotomous outcomes**

| **Outcome** | **Control group, n/N (%) [95% CI]** | **Intervention group, n/N (%) [95% CI]** | **Absolute risk difference, percentage pointsa (95% CI)** | **Relative riskb (95% CI)** | **Odds ratioc (95% CI)** |
| --- | --- | --- | --- | --- | --- |
| PM | 18/61 (29.5%) [19.6% to 41.9%] | 4/61 (6.6%) [2.6% to 15.7%] | -23.0 (-36.0 to -9.4) | 0.22 (0.08 to 0.62) | 0.17 (0.05 to 0.53) |
| VAP | 16/61 (26.2%) [16.8% to 38.4%] | 3/61 (4.9%) [1.7% to 13.5%] | -21.3 (-33.9 to -8.6) | 0.19 (0.06 to 0.61) | 0.15 (0.04 to 0.53) |
| MDR-PI | 12/61 (19.7%) [11.6% to 31.3%] | 3/61 (4.9%) [1.7% to 13.5%] | -14.8 (-26.8 to -3.0) | 0.25 (0.07 to 0.84) | 0.21 (0.06 to 0.79) |
| UE | 19/61 (31.1%) [20.9% to 43.6%] | 2/61 (3.3%) [0.9% to 11.2%] | -27.9 (-40.5 to -15.0) | 0.11 (0.03 to 0.43) | 0.07 (0.02 to 0.34) |

**Panel B. Continuous outcomes**

| **Outcome** | **Control group, mean ± SD** | **Intervention group, mean ± SD** | **Mean differenced (95% CI)** |
| --- | --- | --- | --- |
| Duration of mechanical ventilation (hours) | 72.33 ± 47.79 | 80.67 ± 72.20 | 8.34 (-13.64 to 30.32) |
| ICU length of stay (days) | 19.84 ± 16.65 | 17.89 ± 12.58 | -1.95 (-7.24 to 3.34) |
| Nursing checklist compliance (%) | 67.90 ± 11.05 | 90.80 ± 6.69 | 22.90 (19.62 to 26.18) |

**Table S2. Segmented Poisson Regression Effects of SAFE-CARE on Primary Outcomes per 100 intubation days**

| **Outcome** | **Baseline rate per 100 intubation days (95% CI)** | **Pre-intervention monthly trend, RR (95% CI)** | **Immediate change at first post-intervention time point, RR (95% CI)** | **Relative monthly slope change after intervention, RR (95% CI)** | **Post-intervention monthly trend, RR (95% CI)** | **Pseudo R²** |
| --- | --- | --- | --- | --- | --- | --- |
| PM | 7.36 (2.93 to 18.53) | 1.03 (0.90 to 1.19) | 0.26 (0.05 to 1.35) | 0.82 (0.55 to 1.23) | 0.85 (0.58 to 1.24) | 0.43 |
| VAP | 6.69 (2.52 to 17.73) | 1.03 (0.88 to 1.19) | 0.38 (0.06 to 2.20) | 0.58 (0.25 to 1.37) | 0.60 (0.26 to 1.38) | 0.47 |
| MDR-PI | 5.36 (1.76 to 16.29) | 1.01 (0.85 to 1.21) | 0.54 (0.08 to 3.58) | 0.59 (0.25 to 1.39) | 0.60 (0.26 to 1.38) | 0.36 |
| UE | 8.37 (3.45 to 20.30) | 1.02 (0.89 to 1.17) | 0.31 (0.05 to 2.13) | 0.37 (0.06 to 2.13) | 0.37 (0.06 to 2.15) | 0.61 |

**Notes:** Values are presented as rate ratios (RRs) from segmented Poisson regression models with a log link and log(intubation days) as an offset, except for the baseline rate, which is expressed per 100 intubation days. Under the P1 parameterization, the immediate intervention effect at the first post-intervention time point was calculated as exp(β2 + β3), and the post-intervention monthly trend as exp(β1 + β3). Pseudo R² (Cox–Snell) is reported in place of adjusted R².

**Figure S1. ITS plots of the four airway-related outcomes.** (a) PM rate; (B) VAP rate; (C) VAP rate; and (D) UE rate.

**
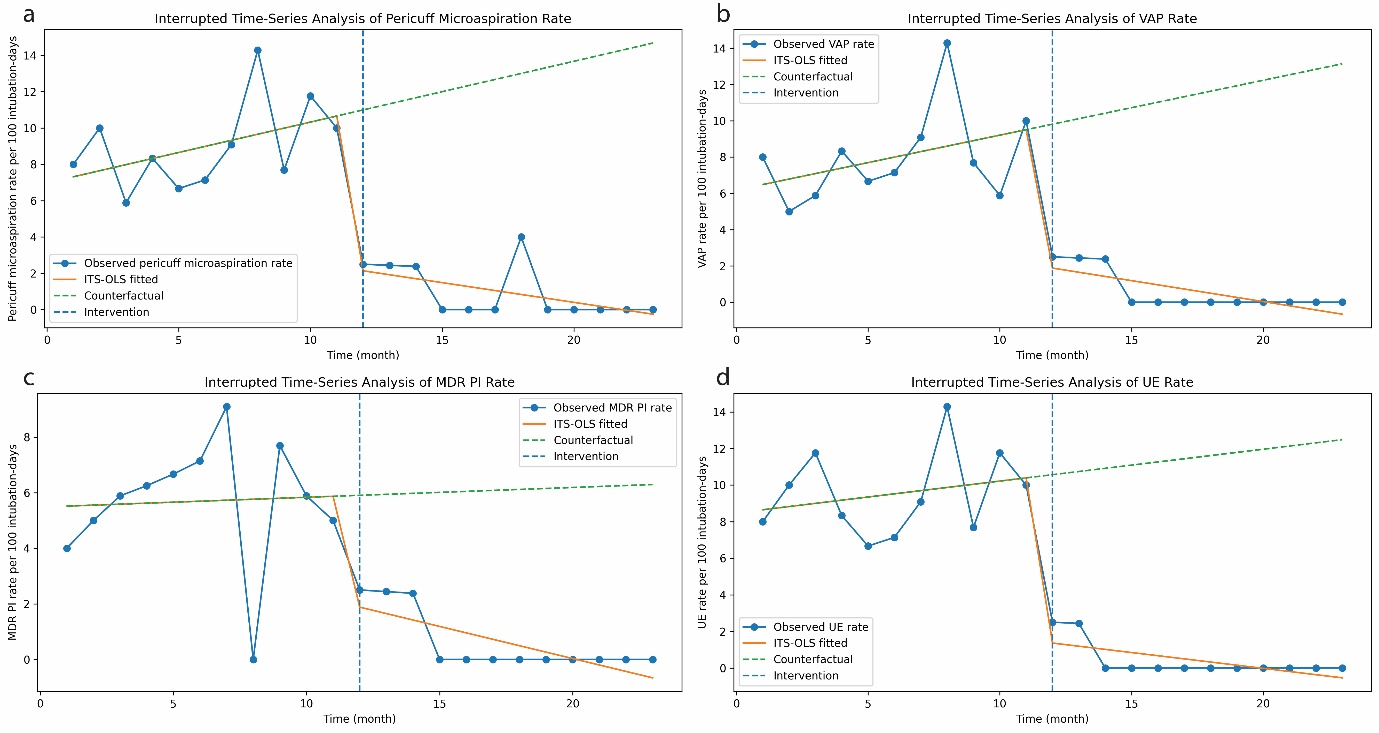
**

**Figure S2. ACF plots of residuals from the ITS-OLS models. (a) PM, (b) VAP, (c) MDR PI, and (d) UE.
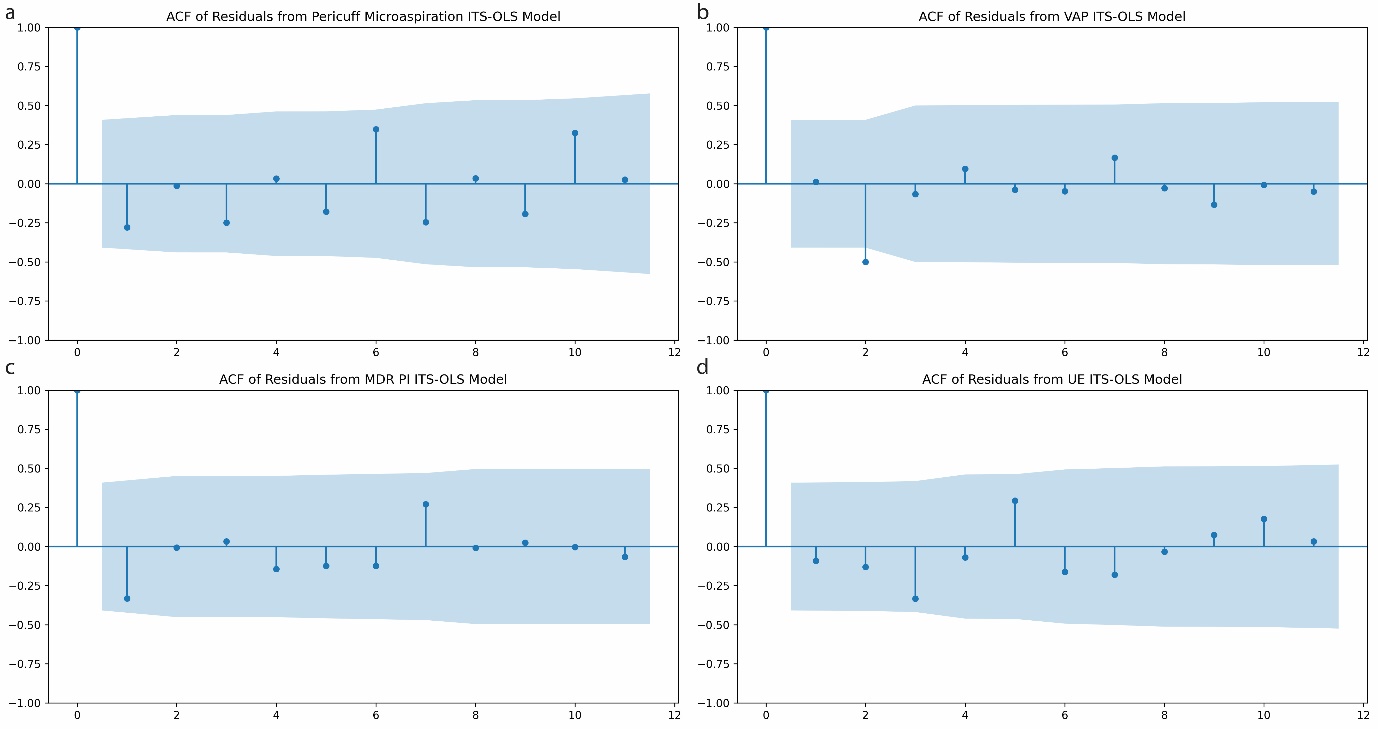
**
